# Draft Genome Sequences of Five Methicillin-Sensitive Staphylococcus aureus Isolates from Skin Lesions in Patients with Atopic Dermatitis in the Russian Federation

**DOI:** 10.1128/MRA.00879-18

**Published:** 2018-10-04

**Authors:** Olga Dmitrenko, Sergey Alkhovsky, Anna Balbutskaya, Timur Tikhomirov, Natalia Fedorova, Alexander Gintsburg

**Affiliations:** a Department of Bacteriology, N. F. Gamaleya National Research Center for Epidemiology and Microbiology, Ministry of Health of Russian Federation, Moscow, Russia; b Laboratory of Biotechnology, D. I. Ivanovsky Institute of Virology, N. F. Gamaleya National Research Center for Epidemiology and Microbiology, Ministry of Health of Russian Federation, Moscow, Russia; c Federal Research Center All-Russian Scientific Research Institute of Experimental Veterinary Medicine Named after K. I. Skryabina and Y. R. Kovalenko, Russian Academy of Sciences, Moscow, Russia; d Department of Dermatovenereology of Pediatric Faculty, Pirogov Russian National Research Medical University, Ministry of Health of Russian Federation, Moscow, Russia; e Laboratory of Clinical Bacteriology, Pirogov Russian National Research Medical University, Ministry of Health of Russian Federation, Moscow, Russia; f Department of Genetics and Molecular Biology of Bacteria, N. F. Gamaleya National Research Center for Epidemiology and Microbiology, Ministry of Health of Russian Federation, Moscow, Russia; Loyola University Chicago

## Abstract

We present here draft genome sequences of five Staphylococcus aureus strains obtained from children suffering from atopic dermatitis. The strains were determined to be of five different sequence types (sequence type 1 [ST1], ST7, ST8, ST15, and ST101) and carried a unique combination of superantigen-like protein (SSL) and serine protease genes.

## ANNOUNCEMENT

Atopic dermatitis (AD) is a chronic inflammatory skin disease. The incidence of AD in children in developed countries has reached 20% (1). Colonization by superantigen-producing Staphylococcus aureus is an important aggravating factor of AD and recurrent skin infection (2, 3). Our previous investigation of S. aureus clinical isolates (obtained from Columbia blood agar) determined that about 30% of isolates did not contain superantigen toxin genes (4). A total of 5 S. aureus strains from patients with a high SCORing Atopic Dermatitis (SCORAD) index and without superantigen genes were selected for whole-genome sequencing to assess their pathogenic traits. All strains were subcultivated in brain heart infusion broth (BD Diagnostic Systems) and incubated overnight at 37°C. DNA samples were extracted using a modified protocol (BioSilica, Russia). DNA libraries were prepared with a New England Biolabs NEBNext Ultra DNA library prep kit for Illumina according to the manufacturer's protocol. Whole-genome sequencing was performed using the Illumina HiSeq platform with coverage of 250 to 300× for each genome. *De novo* assembly was performed using CLC Genomics Workbench v.7.0. The results are presented in Table 1. The genome sizes ranged from 2.66 to 2.78 Mb, with a G+C content of 32.7%. Annotation was carried out using the Rapid Annotation using Subsystems Technology (RAST) server (http://rast.nmpdr.org ) (5 ) and the NCBI Prokaryotic Genome Annotation Pipeline (PGAP) (https://www.ncbi.nlm.nih.gov/genome/annotation_prok/). All strains were genotypically characterized by multilocus sequence type (MLST) analysis (https://cge.cbs.dtu.dk/services/MLST) (6) and *spa* typing ( https://www.spaserver.ridom.de).

**TABLE 1:** Strain-identifying information and basic statistics for draft genome sequences

S. aureus strain^*a*^	MLST	*spa* type	GenBank accession no.^*b*^	Total size (bp)	No. of contigs >1,000 bp	No. of genes		No. of RNA genes	
						Total	Coding	RNAs	tRNAs
0780-1302-2015	ST101	7170	PQWV00000000	2,782,298	15	2,834	2,690	65	56
0257-2201-2015	ST15	2398	PQWU00000000	2,666,594	52	2,763	2,622	70	59
0345-2701-2015	ST8	024	PQWT00000000	2,742,926	46	2,886	2,742	69	59
0908-2002-2015	ST1	127	PQWS00000000	2,754,974	43	2,896	2,745	70	59
1014-2602-2015	ST7	091	PQWR00000000	2,713,020	79	2,851	2,713	67	58

a Each strain had one intact prophage.

b The raw sequences of the *S. aureus* strains have been deposited in the SRA database under BioProject number SRP159856.

Prophage regions were revealed using PHASTER (7), corrected by RAST (precise localization in the genome) and CLC (attachment sites). Cytotoxin-coding genes were identified as the following: alpha-hemolysin, gamma-hemolysin HlgAB/HlgCB, hemolysin III, leukocidins (LukED and LukGH), delta-hemolysin, and alpha and beta classes of phenol-soluble modulins. Genes coding the superantigen-like protein (SSL) family and serine proteases were determined. The results were confirmed by BLASTp. All strains carried beta-converting prophages with immune evasion cluster genes for staphylokinase and chemotaxis inhibitory protein, as well as a metallo-beta-lactamase gene. The sizes of prophage genomes varied from 42,735 to 43,758 bp, with G+C contents of 33.0 to 33.5%. No plasmids were identified using PlasmidFinder (8). The strains were determined to be of the five ST that are the most prevalent among invasive S. aureus strains in Europe (6, 9). The strains, which were isolated from patients with chronic staphylococcal infection, contained a unique combination of SSL and serine protease genes.

A more detailed report from a full comparative genomic analysis will be included in future publications.

### Data availability.

The GenBank accession numbers for these five genome sequences (PQWV00000000, PQWU00000000, PQWT00000000, PQWS00000000, PQWR00000000) are listed in Table 1.
